# Effects of glucocorticoids in depression: Role of astrocytes

**DOI:** 10.3934/Neuroscience.2018.3.200

**Published:** 2018-08-28

**Authors:** Pranav Chintamani Joshi, Sugato Benerjee

**Affiliations:** Department of Pharmaceutical Sciences and Technology, Birla Institute of Technology, Mesra, Ranchi, India

**Keywords:** anxiety, astrocytes, depression, glucocorticoids, stress

## Abstract

Astrocytes or astroglia are heterogeneous cells, similar to neurons, that have different properties in different brain regions. The implications of steroid hormones on glial cells and stress-related pathologies have been studied previously. Glucocorticoids (GCs) that are released in response to stress have been shown to be deleterious to neurons in various brain regions. Further, in the light of the effect of GCs on astrocytes, several reports have shown the crucial role of glia. Still, much remains to be done to understand the stress-astrocytes-glucocorticoid interactions associated with the pathological consequences of various CNS disorders. This review is an attempt to summarize the effects of GCs and stress on astrocytes and its implications in depression.

## 1. Introduction

Astrocytes or astroglia are highly heterogeneous glial cells that populate the brain and spinal cord, and are responsible for homeostasis of the central nervous system (CNS). Earlier, it was reported that, about 90% of the cortical tissue volume is made up of astrocytes, whereas the remaining 10% consists of neuronal cell bodies and blood vessels in the rat cerebral cortex [1]. However, the cellular composition of mammalian brain showed that astrocytes are only 20% of its glial cells; the majority (75%) are oligodendrocytes, while microglia amount to only 5% of glial cells in the grey matter [2]. Astrocytes, representing a large glial population in the mammalian brain [3], play a pivotal role in contributing to the brain metabolism [4] and are involved in glutamate clearance from the synapse and cycling of glutamine back into neurons (e.g. Glutamate-glutamine metabolism and transport) [5]. Astrocytes also release gliotransmitters such as glutamate and ATP [6], both onto neurons and other glial cells [6]. In contrast to chemical coupling at the synapse, astrocytes have extensive coupling via gap junctions, forming dynamic networks that passage molecules between astrocytes as well as to other cells in the CNS [7]. This passage includes interactions with the blood-brain barrier (BBB), mainly the vascular endothelial cells, as well as transporting metabolites to supply the energy needs of neurons [7],[8].

The adrenal gland which secretes Glucocorticoids (GCs), in response to the signal from the hypothalamus, is responsible for producing a range of effects in response to stress. The release of GCs, a class of steroid hormones, is regulated by the hypothalamic-pituitary-adrenal (HPA) axis [9]. The HPA axis that belongs to neuroendocrine systems is commonly associated with stress signaling and the “fight-or-flight” response [10]. GCs act as potent transcriptional regulators that signal through two types of receptors: The high-affinity mineralocorticoid receptors (MRs) and the lower affinity glucocorticoid receptors (GRs) [11].

The MRs are associated with mechanisms involving lower GC concentrations, while the GRs respond to higher concentrations of GCs, such as levels associated with stressful experiences [12]. Alterations in the GC signaling are thought to contribute to disorders like Major Depression (MD) [13], Alzheimer's disease [14], and Cushing's syndrome [15], etc. A recent study on MD, showed that reduced levels of glial fibrillary acidic protein (GFAP) immunoreactive astrocytes [16] while the release of GCs in response to chronic stress leading to MD has been widely reported [17]. However, the role of stress hormones on astrocytic gene expression patterns, remain largely uncharacterized. Astrocytes are involved in primary biochemical processes and are one of the primary cell types in the brain [18]. An attempt is being made to explore the role of astrocytes in neuropsychiatric disorder like depression [19]. In this review, we primarily focus on the effects of the stress-induced GCs on the astrocytes and their implications in depression.

## 2. Astrocytes and depression

Initially, depression was viewed as a neuron-based disorder. This “neuron-centric” view of depression is at least partially supported by the known actions of commonly used antidepressants that modulate levels of the neurotransmitters in the brain (e.g. Serotonin by SSRIs) [20]. However, antidepressants target proteins expressed in multiple brain cell types (e.g. astrocytes express NMDA receptors and serotonin receptors [21]), thus distinct mechanisms and cells involved in antidepressant action remain largely unknown. A series of studies in the late 1990s and early 2000s made unexpected associations between depression and changes in cell density of specific cell types in the brain. Rajkowska and colleagues used cell counting approach to investigate how depression impacted cell density and morphology across various brain regions. Based on previous models, they suspected that depression would lead to cell death and decreased cell density in brain regions associated with mood disorders. They found that there were indeed decreases in cell density in areas such as the prefrontal cortex (PFC) [22] and hippocampus [23]. But, their findings were surprising regarding the cell type; the decreases in cell density were associated with morphologies consistent with both neurons and glial cells [24].

Psychiatric disorders like schizophrenia, bipolar disorder and MD involve reduced astrocytes and astroglial atrophy [25]. Postmortem studies of depressed patients showed reduced glial cell densities in the PFC, amygdala and hippocampus [24]. Banasr and Duman demonstrated that the depressive behavior can be induced by chemical astrocytes ablation in the PFC of rat [26]. Moreover, hippocampal astrocytic loss is associated with chronic stress [27] and also chronic stress interferes with glial cell metabolism via glutamatergic mechanisms [28]. It remains unclear as to how dysregulated GCs may be involved in the loss of astrocytes after stress.

**Figure 1.**
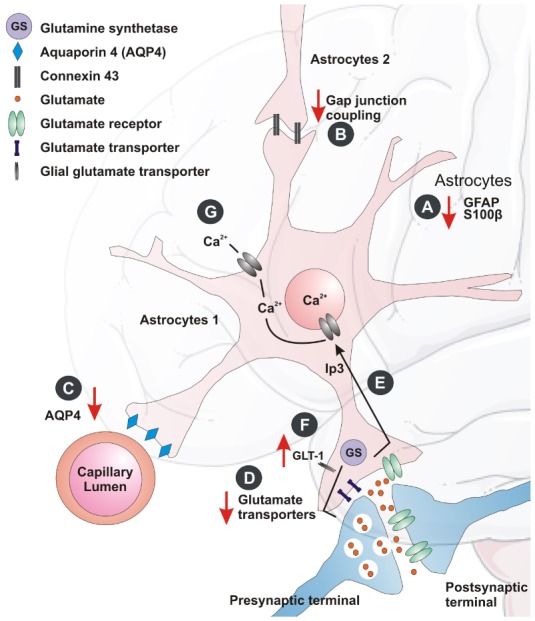
Astrocytic changes in the depressed brain. The crosstalk between the astrocytes and neurons is mediated at the tripartite synapse: A) Astrocyte marker GFAP and cytoplasmic protein S100β expression may alter with depression. B) Astrocyte-specific gap junction protein (Cx43) may alter during stress and depression. C) The water channel marker, AQP4 protein levels is found to reduce in depression. D and E) The expression of certain astrocyte glutamate transporters, as well as glutamine synthase may reduce in MD patients. F) There may be up-regulation of the hippocampal glial glutamate transporter (GLT-1) after chronic stress. G) Astrocytes may also coordinate their function through Ca^2+^ excitability and its downstream signaling in depression.

These findings inspired biochemical studies that explored specific depression-linked decrease in astrocyte density based on reduced protein expression of GFAP (a biomarker for mature astrocytes). GFAP has shown its significant role in pathogenesis of various CNS pathologies [29]. The GFAP expression studies, showed a 15% loss of total astrocyte volume and decrease in expression in the brains of depressed individuals [24],[30], while expression in patients increased after treatment with antidepressants. A different investigation on postmortem amygdale tissue from depressed patients showed reduction in nissl positive astrocytes [31]. Another astroglial marker, the calcium binding protein S100β was reported to be a marker of minor depression [32]. S100β was found to be reduced in the ventral PFC of depressed suicide victims (Figure 1A). The screening of 36 biological markers in 30 inbred mouse strains [33] showed that GFAP, S100β , glyoxalase 1 and histone deacetylase 5 responded to chronic treatment with fluoxetine. Similarly, reduction in the astroglial GFAP-positive cells and its overall immunoreactivity were detected in several animal models of chronic stress [24]. In situ hybridization and RT-qPCR gene expression studies with human brain samples allowed the detection and quantification of the mRNA present in brain sections and showed alterations in several transcripts for astrocyte proteins with depression in the locus coeruleus [24],[34]. They include astrocytic markers like S100β, GFAP (Figure 1B), gap junction proteins (gap junction protein alpha 1 and 6 (Gja1 and Gja6)), and membrane channels proteins (Aquaporin-4 (AQP4)). It was also found that the reduction of glutamate transporter (GLT-1), glutamate aspartate transporter (GLAST), glutamine synthetase (GS), connexins (Cx43 and Cx30) [35],[36] (Figure 1C). Similarly, the reports show a decrease in the mRNA expression of GLT-1, GLAST, and GS in the anterior cingulate and dorsolateral PFC [37], while some of these transcripts were reduced in other brain areas. This suggests that the astroglial cells may contribute towards altered neuroglial networks in different forms of depression.

In animal models, selective ablation of astroglial cells (L-a-aminoadipic acid, which envenoms astrocytes) triggered depressive behavior [24]. Pharmacological inhibition of astroglial gap junction connectivity [38] or astroglial plasmalemma glutamate transporters resulted in anhedonia [39] (Figure 1F), one of the key symptoms of depression. All these findings in animals subjected to depression models indicate astrocytic abnormality. After chronic exposure to chronic unpredictable stress (CUS), it was found an increase in glutamate release and reduced uptake in the hippocampus [40]. In the learned helplessness model, a similar trend of reduced glutamate uptake was also observed in the PFC, striatum and hippocampus [41]. Studies in rats also showed blockage of astroglial glutamate uptake and was sufficient to induce anhedonic state marked by decreased sucrose consumption [42]. Ketamine and riluzole, which acts as glutamate modulators, have shown antidepressant effects in patients and animal models [43].

Concurrent pathophysiology showed aberrant glutamatergic neurotransmission as a primary mechanism for major psychiatric disorders, including MD [44]. Astrocytes are fundamental elements in glutamatergic and GABAergic neurotransmission being the hubs for glutamate—glutamine and glutamine—GABA shuttles [45]. In the brains of MD patients, expression of astrocyte-specific glutamate transporters GLT-1, GLAST as well as glutamine synthase (GS) (Figure 1D, E) are reduced [24], indicating compromised astrocytic uptake of glutamate, as well as decreased glutamine production.

Interactions between glial and neuronal cells may be impaired at synaptic level in depression-like conditions. Purines may modulate glia-neuron communication bi-directionally, restoring the synaptic efficacy and reversing depression-like behavior [46]. Another possible trigger for depressive episodes was the cytokine signaling in astrocytes [47]. Recent research focused on an alternative form of neuro-glial signaling, namely, secreted extracellular vesicles (EVS) [48]. The EVS, such as ectosomes and exosomes, capable of carrying mRNA and microRNAs (miRNA) have shown a role in intercellular signaling, and may also underlie depressive behavior. The transfer of exosomes to neurons is mediated through oligodendrocytes, microglia and astrocytes, which may either support neurons or promulgate the disease [48]. Thus neuroglia communication at the synaptic level is far from clear and warrants further research.

The mechanism underlying antidepressants action of fluoxetin is mediated by regulating astrocytic AQP4 levels that affects astrocyte morphology and further restore the functional glia-vasculature interface [48]. The decreased astrocyte-specific Cx43 levels, as stated earlier, appear to be related to antidepressant and anxiolytic phenotypes [49]. In accordance, the antidepressants show an intricate pattern linking astrocytes and connexins to address the mechanisms of action of these compounds [50]. Thus the role of astrocytes in depression is far from clear and needs further work.

## 3. Mechanisms associating stress, GCs, and astrocytic functions

The postmortem studies on humans and animals showed reduction in astrocyte density and function in the limbic regions of the brain suggest probable mechanism contributing to pathology of stress and GCs overproduction [51]. Indeed, a selective volume reduction of hippocampus and PFC was observed following chronic stress [51]. The neuronal proliferation in the dentate gyrus (DG) [52] was decreased after corticosterone treatment and psychosocial stress while the postmortem studies in patients with a history of high-dose steroid treatment. However, there was no reduction in the number of neurons [53]. This suggests that the volume reduction cannot be entirely due to stress-induced reduction of neurogenesis. The increased GCs immunoreactivity was observed in astrocytes of amygdala in depressed patients compared to healthy controls or bipolar disorder patients [54], suggesting that astrocytes may respond to changes in stress hormone levels.

Studies on chronic stress may result in reduced gliogenesis in the hippocampus and PFC [23],[28] while *in-vitro* studies showed the dexamethasone (a synthetic glucocorticoid) blocking astrogliogenesis from neural precursor cells. These findings suggest that, the perturbed astroglial cells in stress is likely to contribute in region-specific volume changes commonly observed in stress-related pathologies [55].

The role of astrocytes in neurodegenerative diseases and inflammatory processes is well documented [56]. It is well reported that human postmortem tissue in major depressive disorders (MDD) have shown alterations in the expression of mRNA and protein for astrocyte markers such as GFAP, Cx40, Cx43, AQP4, S100β and glutamatergic markers including GLT-1, GLAST, and GS [53]. When the brain gets injured, astrocytes become activated. This is characterized by changes in the gene expression profiles and high levels of GFAP [57]. In addition to this, exposure to corticosterone for short (6–24 h) and prolonged (3 weeks) period in astrocytic cultures show an increase in GFAP mRNA levels and a decrease when astrocytes were co-cultured with neurons [57]. This supports the findings that GCs may also regulate the synthesis of GFAP. Further, it also suggests the crosstalk between neuron and astrocytes to withstand deleterious effects of corticosterone. Initial *in-vivo* studies on rats have shown decreased hippocampal and cortical GFAP mRNA levels [58] while immunohistochemical studies have shown increased GFAP immunoreactivity in a dose and brain-region specific manner following chronic corticosterone treatment [59]. The complex nature of corticosteroids regulating GFAP during stress can be understood wherein 6 days of stressful activity leads to 30% increase in hippocampal GFAP-immunoreactive astrocytes [60] and at the same instance, the studies on adult rats exposed to early-life stress showed reduced density of GFAP-immunoreactive astrocytes in various limbic regions of brain [61]. This GFAP binary response may suggest a primary astrocyte-mediated neural protection, which may subsequently turn neurotoxic depending on the dose, which will affect the brain region as well as the time of exposure to stress.

Astrocytes express most of the receptors and additionally, ion channels found in neurons are involved in recycling and eliminating glutamate from synapses, thereby contributing to glutamatergic synaptic transmission [62]. The glial transporters help glutamate uptake and conversion to glutamine with the help of enzyme glutamine synthase (GS), which is responsive towards GCs [63]. A current hypothesis states that excessive extra synaptic glutamate leads to the atrophy of apical dendrites seen in hippocampal pyramidal neurons in stressed rats [64]. Studies aiming to understand the regulation of GS have shown glucocorticoid-mediated regulations of glial GS during stress [63]. Likewise, chronic stress showed up-regulation of the hippocampal glial glutamate transporter (GLT-1) (Figure 1F) [65]. This suggests that glutamate cycling is regulated by corticosteroids by induction of GS and GLT-1 expression in a time-dependent manner in distinct subpopulation of astrocytes. The hypothesis that GCs inhibit glucose uptake was substantially evidenced by a study in which it was shown that chronic mild stress exacerbates the consequences of chronic cerebral hypoperfusion [66]. This results in a lack of energy to neurons and astrocytes for high-affinity glutamate reuptake and thus an increase in vulnerability of the brain [67].

Astrocytes lie in proximity of BBB and play an important role in its maintenance by interacting with endothelial cells. On this regard, it would be important to emphasize that modulation of the barrier “tightness” is a result of complex interaction between GCs and cells of neurovascular units including astrocytes [68].

As stated earlier, S100β found primarily in the cytoplasm of astrocytes, and is involved in glia-neuron signaling [69]. The S100β regulates a variety of intra and extracellular functions such as cell growth, metabolism, calcium homeostasis and synaptic plasticity [69]. This suggests the possible role of astrocytes in regulating neuronal synaptic plasticity. Furthermore, reports suggest that S100β concentration was reduced after maternal administration of betamethasone (a synthetic glucocorticoid) in the hippocampus and serum of the neonate rat [70]. This suggests that increased GCs or chronic stress may reduce the expression or function of S100β. In an another study, stress exposure increased S100β concentration in cerebrospinal fluid (CSF) after acute predator stress [71] and chronic restraint stress [72]. These findings reveal the biphasic response of S100β to stress. This observation was also supported by another study where the astrocyte cultures were exposed to dexamethasone (Table 1)[73].

**Table 1.** Different markers that are expressed in Astrocytes in response to GCs stimuli.

Name of the marker	Function	References
Glial Fibrillary Acidic Protein (GFAP)	Cytoskeletal protein	[58]
S100β	astroglia-specific neurotrophic factor	[82],[83]
Nerve growth factor (NGF)	Neurotrophic factor	[73]
Basic fibroblast growth factor (bFGF)	Neurotrophic factor	[73]
N-myc downstream- regulated Gene (Ndrg2)	Cell differentiation factor	[84]
Glutamine synthetase (GS)	Recycling of the glutamate	[37],[53],[63]
Glial glutamate transporter (GLT-1)	Recycling of the glutamate	[37],[53],[63]

Astrocytes may coordinate their function through Ca^2+^ excitability and subsequent signaling that have also been implicated in depressive disorders [74]. Surprisingly, astrocytic calcium signaling is regulated by GCs [75]. This apparently suggests the role of GCs in the modulation of glial calcium cell signaling during depression and anxiety. Additionally, the GCs are critical regulators of brain development and brain aging. Rat astrocyte primary culture study showed that dexamethasone increases intracellular and membrane-associated lipocortin-1 (annexin-1) [76] while it increased and reduced expression of nerve growth factor (NGF) in cultured neurons and astrocytes respectively [77].

Recent work focused on how the astrocytes intervene in the GC-induced stress and its probable mechanism. It has been shown that 5' AMP-activated protein kinase (AMPK) may mediate down regulation of GRs in astrocytes of rat PFC [78]. Also, GRs in astrocytes, as a critical stress-responding transcriptional factor, may mediate stress-induced adaptation via regulating the expression of astrocyte-derived neurotrophic factors. Researchers also found that the chronic mild stress-induced decrease in brain-derived neurotrophic factor (BDNF) and Cx43 causing astrocytic dysfunction by forming abnormality in gap junctions in the PFC of rats [79],[80]. Some recent studies in emotional processing show astrocytes' crucial role, along with neurons, in the hippocampus, PFC, and amygdala [81]. Thus current astrobiology research suggests an evolving role of neuro-astroglial communication in the modulation of depression, opening new avenues of therapies against depressive disorders.

## 4. Conclusion

The studies over recent years have started unveiling the role of astrocytes in stress and their response to GCs release thus shifting our understanding from neuron-centric theories towards the role of these glial cells in the neurobiology of stress. However, the role of released GCs in response to stress and its effects on astrocytes need to be explored further in for a better understanding of stress- or glucocorticoid- related brain disorders.
